# Docking-Based Evidence for the Potential of ImmunoDefender: A Novel Formulated Essential Oil Blend Incorporating Synergistic Antiviral Bioactive Compounds as Promising Mpro Inhibitors against SARS-CoV-2

**DOI:** 10.3390/molecules28114296

**Published:** 2023-05-24

**Authors:** Ayoub Ksouri, Anis Klouz, Balkiss Bouhaouala-Zahar, Fathi Moussa, Mounir Bezzarga

**Affiliations:** 1 Laboratoire des Biomolécules, Venins et Applications Théranostiques, Institut Pasteur de Tunis, Université Tunis El Manar, 13 Place Pasteur, BP74, Tunis 1002, Tunisia; balkiss.bouhaouala@fmt.utm.tn; 2 Faculté de Médecine de Tunis, Université Tunis El Manar, 15 Rue Djebel Lakhdhar, La Rabta, Tunis 1007, Tunisia; anis.klouz@rns.tn; 3 Institute of Physical Chemistry, CNRS—UMR 8000, University Paris—Saclay, Rue Noetzlin, 91190 Gif-sur-Yvette, France; 4 Laboratoire de Modélisation Mathématique, Faculté des Sciences de Tunis, Université Tunis El Manar, Analyse Harmonique et Théorie du Potentiel, Campus Universitaire, Tunis 1068, Tunisia; mounir.bezzarga@yahoo.fr; 5 Institut Préparatoire aux Etudes d’Ingénieurs de Tunis, Université de Tunis, Tunis 1068, Tunisia

**Keywords:** ImmunoDefender, essential oils (Eos), bioactive molecules, antiviral, SARS-CoV-2, main-protease, active and allosteric sites

## Abstract

Essential oils (Eos) have demonstrated antiviral activity, but their toxicity can hinder their use as therapeutic agents. Recently, some essential oil components have been used within safe levels of acceptable daily intake limits without causing toxicity. The “ImmunoDefender,” a novel antiviral compound made from a well-known mixture of essential oils, is considered highly effective in treating SARS-CoV-2 infections. The components and doses were chosen based on existing information about their structure and toxicity. Blocking the main protease (Mpro) of SARS-CoV-2 with high affinity and capacity is critical for inhibiting the virus’s pathogenesis and transmission. In silico studies were conducted to examine the molecular interactions between the main essential oil components in “ImmunoDefender” and SARS-CoV-2 Mpro. The screening results showed that six key components of ImmunoDefender formed stable complexes with Mpro via its active catalytic site with binding energies ranging from −8.75 to −10.30 kcal/mol, respectively for Cinnamtannin B1, Cinnamtannin B2, Pavetannin C1, Syzyginin B, Procyanidin C1, and Tenuifolin. Furthermore, three essential oil bioactive inhibitors, Cinnamtannin B1, Cinnamtannin B2, and Pavetannin C, had significant ability to bind to the allosteric site of the main protease with binding energies of −11.12, −10.74, and −10.79 kcal/mol; these results suggest that these essential oil bioactive compounds may play a role in preventing the attachment of the translated polyprotein to Mpro, inhibiting the virus’s pathogenesis and transmission. These components also had drug-like characteristics similar to approved and effective drugs, suggesting that further pre-clinical and clinical studies are needed to confirm the generated in silico outcomes.

## 1. Introduction

The COVID-19 pandemic has caused global human health threats, resulting in a large number of infections, severe forms of the disease, long-term health consequences, and high mortality rates, especially among older and vulnerable populations [1,2,3,4]. As an obligate intracellular pathogen, the SARS-CoV-2 virus requires entry into host cells to multiply, and the crucial role of the non-structural protein nsp5 or main protease in the virus’s life cycle has been identified [5,6,7]. Since SARS-CoV-2 Mpro has no human homolog, it is considered an ideal target for antiviral treatments [8,9,10].

The development of new drugs against COVID-19 is a time-consuming and expensive process, and natural sources have been considered a crucial therapeutic approach. Recent studies have suggested that herbal extract substances and essential oils possess antiviral, anti-inflammatory, and immunomodulatory properties [11,12,13,14].

Additionally, using a blend of essential oils can provide a broader range of therapeutic benefits, as each oil may have unique properties that can target different aspects of the condition being treated. By using a mixture of 10 essential oils, we can potentially achieve a more well-rounded and effective treatment approach compared to using just a few oils.

Furthermore, the use of multiple essential oils in a blend is a common practice in aromatherapy and has been shown to be safe and effective. Studies have shown that aromatherapy can have beneficial effects on a range of conditions [5,6,7].

This study investigated the potential of a treatment approach comprising a mixture of essential oils for COVID-19, exploring the interaction of essential oil components with viral key protein targets inside of cells. Essential oils contain a complex mixture of chemical compounds that can act synergistically with each other to enhance their overall efficacy. This synergy is a well-known phenomenon in aromatherapy and is one of the reasons why blends of essential oils are often used to achieve specific therapeutic effects [15].

Essential oils have been found to exhibit broad-spectrum antiviral activity against various viruses, including Herpes simplex virus, Influenza virus, Respiratory syncytial virus, HIV, human rhinovirus, Rotavirus, Norovirus, Dengue virus, Zika virus, and Chikungunya virus. “ImmunoDefender” is a natural product made from a blend of essential oils that are believed to have antiviral properties. Some of the key chemical components of these essential oils include terpenes, such as limonene, pinene, and caryophyllene, as well as phenols, such as thymol and carvacrol, Cinnamtannin B1, Cinnamtannin B2, Pavetannin C1, Syzyginin B, and Procyanidin C1 [16,17,18,19,20,21,22]. These compounds have been shown to have antimicrobial and antiviral effects and are believed to help boost the immune system by stimulating the production of white blood cells. “ImmunoDefender” is a promising natural product for treating viruses due to its potential ability to help prevent viral infections and reduce symptoms of viral illnesses.

The suggested EO components possess antiviral, anti-inflammatory, and immune-stimulating properties and actions, making them ideal candidates for the development of a natural antiviral compound. A mixture of bioactive compounds consisting of small, efficient, and tolerable amounts of essential oils (Eos) is combined with a natural organic excipient to enhance absorption. Sesame oil is a widely utilized natural and non-aqueous excipient in pharmaceutical formulations. Its primary function is to serve as a vehicle for lipophilic drugs and active ingredients.

The major components of each essential oil have been identified, and chemical data and information (molecular properties and drug-like qualities) have been collected and tested for their ability to inhibit the crucial SARS-CoV-2 non-structural protein target Mpro. This study’s findings could potentially contribute to the development of a natural and effective treatment approach for COVID-19.

## 2. Results

The objective of our study was to investigate the potential inhibitory effects of ImmunoDeffender essential oil components on the SARS-CoV-2 viral particle’s non-structural protein Mpro. To achieve this, we performed molecular docking simulations of Mpro with various ligands, including major components of the essential oil, ensuring that each ingredient was accurately dosed within the safe limits of toxicity and acceptable daily intake (ADI). In determining appropriate doses, we carefully considered toxicity data and the required molecular inhibition action, while citing previous studies or literature supporting their use. We also employed a mathematical approach to assign appropriate weights to each compound based on their kinetic effects on the crucial enzymes implicated in the replication of SARS-CoV-2 (Mpro) and the acceptable daily intake of each compound (mathematical matrix data are available upon request from the corresponding author).

Table 1 and S1 in the Supplementary Data (Supplementary Materials) provide a well-described description of the ImmunoDefender formulation. We hope that these additions will provide a better understanding of our study and ensure that the results are replicable, accurate, safe, and effective. 

**Figure 1:**
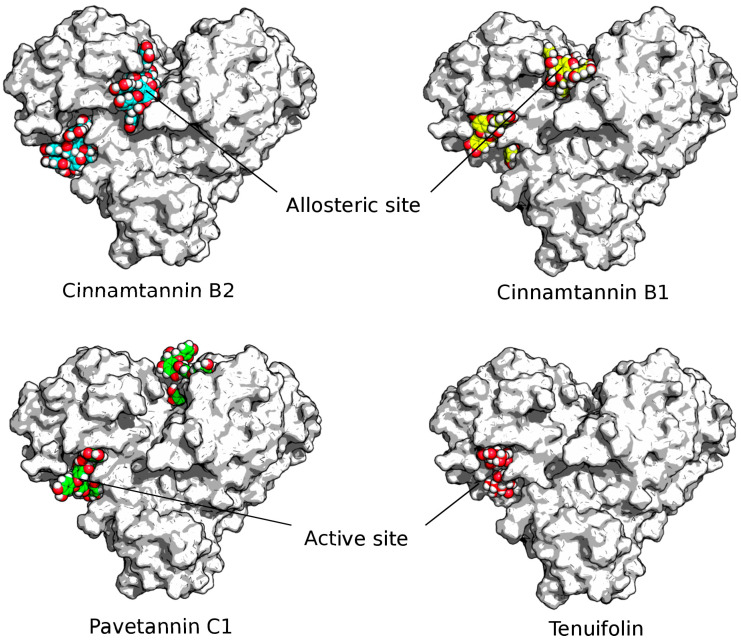
Inhibition of SARS-CoV-2 main protease active and allosteric sites by bioactive ligands. The SARS-CoV-2 main protease (Mpro) is depicted as a gray surface, while Cinnamtannin B1, Cinnamtannin B2, Pavetannin C1, and Tenuifolin are shown (colored by element) as yellow, blue, green, and red spheres, respectively, interacting with both active and allosteric sites. Figure 1 clearly displays the Mpro–ligand complexes, which were visualized using the PyMOL molecular visualizer software (Version 1.3r1) [33]. Notably, several ligands, including Tenuifolin and Procyanidin C1, exhibited remarkable potential for blocking Mpro enzymes, as outlined in Table 2.

This analysis enabled us to pinpoint several bioactive compounds that displayed robust binding affinity for Mpro and exhibited significant inhibitory effects on the key enzymes. These findings suggest the potential utility of these compounds in the development of novel therapeutic options for treating COVID-19. The estimated ΔG (kcal/mol), with which a ligand binds to the active catalytic pocket of the targeted protein and also to the allosteric sites mainly involved in the interaction with the viral polyprotein, was the principal output of the docking carried out using AutoDock Vina as a driven approach and SwissDock as a blind approach for molecular interaction studies. The docking outputs of the ligands are summarized in Table 2 and Table 3 for the Mpro-target protein. For docking data generated via a driven docking based on grid complexes around the active catalytic pockets sites (AutoDock Vina software), complexes with the best conformations of Mpro binding energy were not relevant compared to the results generated using Swissdock software (blind docking against the whole targeted protein structures Mpro; data details are well illustrated in Table S2: Supplementary Date). Complex outputs of Swissdock were visualized, and then, the 3D interaction of the protein–ligand complexes are illustrated in Figure 1.

The proposed essential oil mixture of the ImmunoDefender product has been found to contain 24 bioactive molecule ligands (see Table S1: Supplementary Data). In silico interaction simulations with the target protein Mpor have demonstrated the presence of a significant number of bioactive ligands that interact with high affinity with the proteins. Among these ligands, Cinnamtannin B1, Cinnamtannin B2, Pavetannin C1, Syzyginin B, Procyanidin C1, and Tenuifolin have been shown to interact with remarkable stability and high affinity with the target protein. These ligands are capable of ensuring stable complex conformations via their active catalytic pocket sites, with free binding energies of −9.56, −9.40, −10.30, −10.10, −9.05, and −8.75 (kcal/mol), respectively.

Furthermore, it is noteworthy that nirmatrelvir, the bioactive molecule present in the Paxlovid brand of Pfizer, was tested using the same computational approach of docking. A value of −9.24 was obtained, indicating that the ligands in ImmunoDefender product’s EO mixture have a higher affinity and stability compared to nirmatrelvir. This finding highlights the potential of ImmunoDefender product’s EO mixture in providing therapeutic benefits through its interaction with target proteins.

Intriguingly, the homodimeric structure of the target protein revealed a significant free binding energy in its concave region. Specifically, Cinnamtannin B1, Cinnamtannin B2, and Pavetannin C1 exhibited free binding energies of −11.12, −10.74, and −10.79 (kcal/mol), respectively, within this region. Further analysis showed that these ligands established several hydrogen-bond interactions with specific amino acids of the Mpro group, including Glu14, Asn95, Lys97, Lys100, Pro122, and Asp155 for Cinnamtannin B1, Glu14, Gly15, Ala70, Gly71, Ser121, and Tyr154 for Cinnamtannin B2, and Glu14, Met17, Gly71, Lys97, and Asn119 for Pavetannin C1. This indicates that these ligands possess high affinity and stability towards the target protein and can form stable complex conformations via their active catalytic pocket sites, making them promising candidates for further investigation.

**Figure 2:**
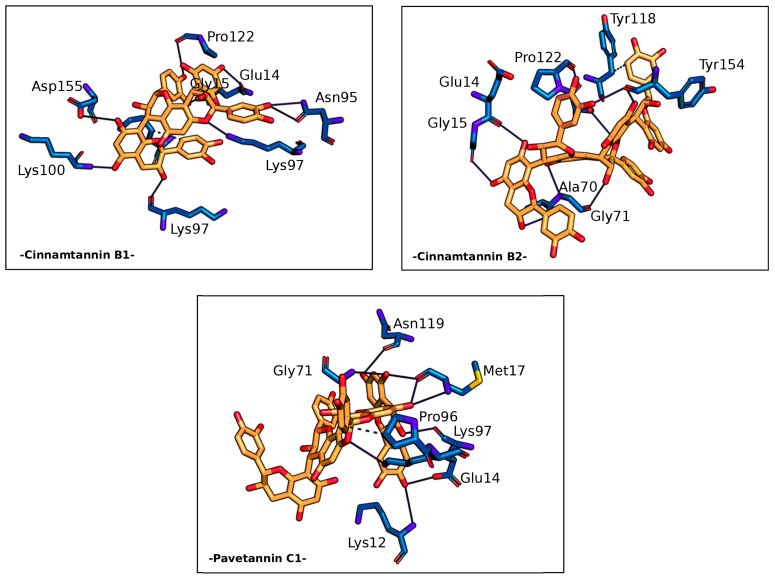
Non-covalent interactions of ImmunoDefender bioactive ligands with allosteric sites in the SARS-CoV-2 main protease. ImmunoDefender bioactive ligands form non-covalent interactions, including hydrogen bonding, with crucial amino acid residues that are involved in the formation of allosteric sites in the SARS-CoV-2 main protease.

Of particular interest, the ligands displayed an exceptionally strong affinity for the Mpro enzyme, as evidenced by the formation of several hydrogen bonds (non-covalent interactions) with crucial amino acid residues in the concave region of the Mpro homodimeric form (Table 3.). This critical interaction region is depicted in detail in Figure 2.

**Figure 3:**
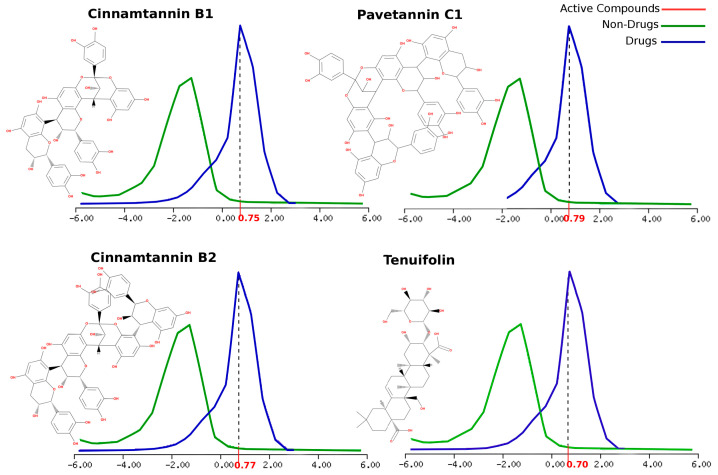
Chemical structures and drug-likeness scores of main ImmunoDefender bioactive compounds. The 2D chemical structures of Main ImmunoDefender bioactive compounds are illustrated, along with their respective drug-likeness scores projected on drug and non-drug graph template graphs. The values described on the *x*-axis are the Drug-Likeness Scores of the bioactive ligands in question provided by Molsoft software.

The main bioactive molecules present in the proposed EO mixture of ImmunoDefender experimental products were carefully selected for further analysis. In order to predict their molecular properties and drug-likeness features, an overall drug-likeness score was computed for each compound based on several molecular property values. As a result, Cinnamtannin B1, Cinnamtannin B2, Pavetannin C1, and Tenuifolin were found to have drug-likeness scores of 0.75, 0.79, 0.79, and 0.70, respectively (as shown in Table 4). Figure 3 illustrates how these score values conform to commercialized drug active molecules with a high level of tolerance and efficiency.

**Figure 4:**
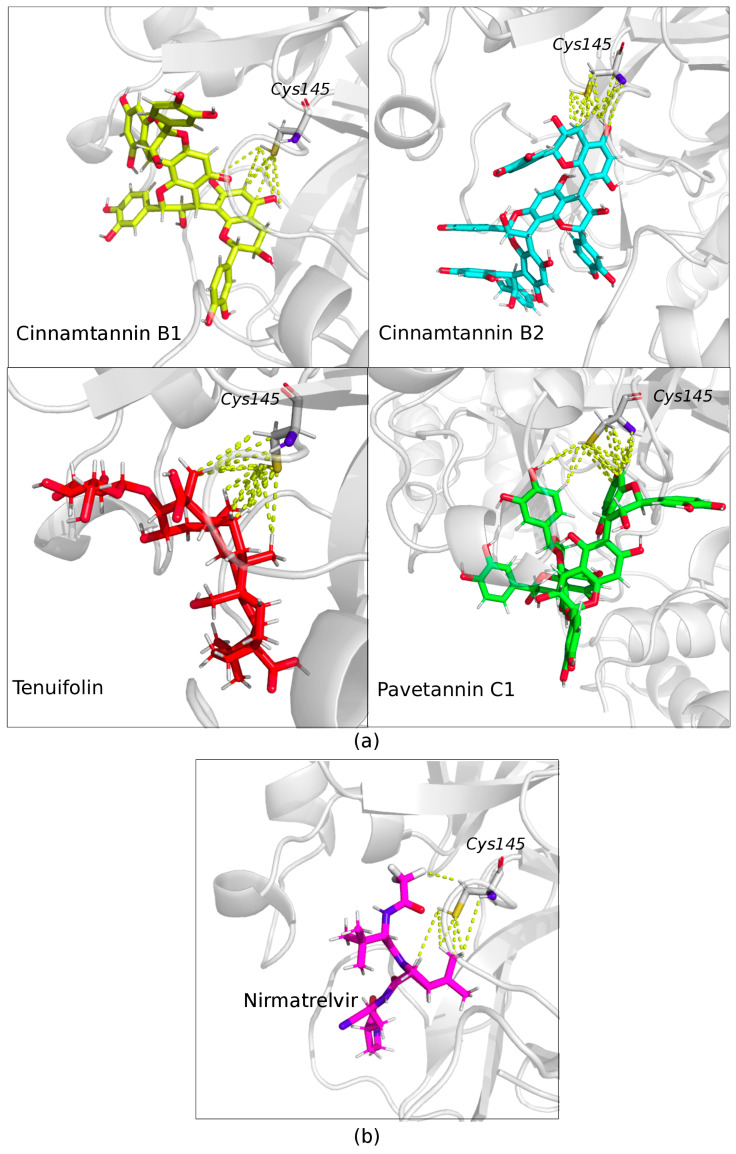
Comparison of binding interactions between ImmunoDefender’s main ligands and Nirmatrelvir, the active component of the anti-COVID-19 drug Paxlovid, with the catalytic pocket of Mpro. The structure of Mpro is depicted as a transparent cartoon, while the ligands and Cys145 residue are shown as sticks. The yellow dashed lines indicate the interactions between the ligands and the catalytic pocket of Mpro. (**a**) ImmunoDefender’s main ligands establish polar, hydrophobic, and hydrogen bond contacts with the catalytic pocket of Mpro. The figure highlights the essential role of the Cys145 residue, which is marked in the figure, in facilitating these interactions. (**b**) Illustration of the interaction between Nirmatrelvir, the chemical ligand of the anti-COVID-19 drug Paxlovid, and the Cys145 residue of Sars-Cov-2 Mpro. This figure underscores the significant contribution of the Cys145 residue to the catalytic activity of Mpro and its potential as a target for the design of anti-COVID-19 drugs.

Interestingly, it was observed that Cystein 145 (Cys145) is the main amino acid responsible for making polar, hydrophobic, and hydrogen bound contacts in the Mpro catalytic pocket between chains, within a distance of 4 ångströms. This observation is illustrated in Figure 4. The presence of these interactions further highlights the potential of the selected bioactive molecules to effectively block the Mpro enzyme activity.

## 3. Materials and Methods

### 3.1. Essential Oil Quantification

The objective of this study was to determine the appropriate doses of essential oils and active constituents used in an antiviral herbal medicinal extract. To ensure the accuracy, safety, and efficacy of our study, we followed a rigorous methodology.

We selected a potent source of the antiviral herbal medicinal extract, which comprised ten pure essential oil substances. The essential oils used were Spearmint *Mentha spicata*, Menthol crystal, Water mint *Menthaaquatica* “*L. (Linnaeus)*”, Cloves *Syzygium aromaticum* “L. *Merr. & L.M. Perry*”, Peppermint *Mentha piperita* “*L. (Linnaeus)*”, Pitch mint *Mentha poulegium* “*L. (Linnaeus)*”, Eucalyptus *Eucalyptus*, Cinnamon *Cinnamomum zeylenicum* “*Blume*”, Cajeput *Melaleuca cajuputii* “*Powell*”, Camphor *Cinnamomum camphora* “*(L.) J. Presl*”, and Sesame *Sesamum indicum* “*L. (Linnaeus)*”. The Supplementary File Table S3 provides a comprehensive and detailed overview of the essential oils of the studied species, making it an excellent resource for those seeking information on this topic.

The quality of the essential oils used in our study was of paramount importance. We took great care to ensure that our selection of essential oils was based on the rate content of active compounds, as specified by the European Pharmacopea, in order to achieve the highest level of purity and potency. Additionally, the source and extraction method of the essential oils were given careful consideration, ensuring that the integrity and quality of the oils were maintained.

To determine the appropriate doses of essential oils and active constituents, we took into account toxicity data and carefully considered the required molecular inhibition action and avoided exceeding toxicity thresholds. We also provided a rationale for the selection of doses, citing previous studies or literature that supported their use. Furthermore, we used an inverse problem of mathematical expectation to determine the appropriate weighting for each compound based on their kinetic effects of temporarily irreversible inhibition of the key enzymes in the replication of the SARS-CoV-2 virus.

Our rigorous adherence to these high standards of selection and dosing ensured the accuracy, safety, and efficacy of the essential oils and active constituents used in our antiviral herbal medicinal extract. This methodology not only allowed us to achieve reliable and reproducible results in our research but also increased the reliability and validity of our findings.

We considered a dose of one gram of bioactive antiviral substances from the essential oils of ten genera of aromatic plants. To determine the appropriate weighting for each compound, we used an inverse problem of mathematical expectation. We assigned weights to each compound based on the kinetic effects of temporarily irreversible inhibition of the key enzymes in the replication of the SARS-CoV-2 virus. We ensured that the accurate ingredient amount combination covers the molecular inhibition action without exceeding toxicity thresholds.

### 3.2. Data Source: Preparation or Protein and Ligands Molecule Files 

In this study, a dataset of bioactive phytochemicals was obtained from the PubChem database [34]; this database contains a wealth of chemical structures, bioactivity, health and safety, and spectra data. The 3D structures were retrieved in the “.sdf” format and converted to “.mol2” format using the OpenBabel web tool [35]. The X-Ray Crystal Structure of the SARS-CoV-2 Main Protease (3C-like protease) was retrieved from the RCSB Protein Data Bank (PDB) [36] with the code PDB ID: 6LU7 [37]. This 3D structure was selected primarily based on its high resolution of 2.16 Å. Since the main protease is functional only in homodimer form, two chains, A and B or a dimeric form, were modeled, starting from the 6LU7 monomer. After removing the N3 inhibitor from the active catalytic pocket of the Mpro, a refinement step was performed using the RELAX application binary executable from the Rosetta package [38]. The protein and ligand structure files were prepared in “.pdbqt” format as follows: hydrogen atoms were added using PDB2PQR (2.1.1 version) software [39] to the query input file (.pdb for receptor & .mol2 for ligands) with a pH of 7.4. The output “.pqr” file was converted to “.pdb” and then to the “.pdbqt” file using the MGLTools (1.5.7 version) software [40].

### 3.3. Receptor-Ligand Docking Process

In order to conduct a thorough analysis of molecular interactions, a comparative docking approach was utilized, employing AutoDock Vina software (version 1_1_2) [41] for blind docking and SwissDock for driven docking. SwissDock [42], which utilizes the EADock DSS docking software (http://www.swissdock.ch) [43], generates a large number of binding modes (BMs) and calculates their CHARMM energies on a grid. The most favorable BMs are ranked based on the FACTS implicit solvation model and then clustered to obtain the most favorable clusters. This process allows for accurate docking assays to be conducted in just a few minutes. For the driven docking approach, active site residues of the main protease were identified using the literature and checked with the molecular visualization system PyMOL. The grid box around these active sites was then set to the active site residues with a spacing of 1 angstrom.

### 3.4. Drug-Likeness Prediction for the Major EO Bioactive Compound

In addition to the molecular docking study, an analysis of the drug-likeness score was conducted using Molsoft software (Version 3.9-3c) [44]. This analysis considered several key molecular properties, including the molecular weight, number of hydrogen bond donors and acceptors, octanol/water partition coefficient, water solubility, molecular polar surface area and volume, pKa of the most basic/acidic group, and the Blood-Brain Barrier score. The drug-likeness chemical fingerprint scores were then projected onto a large-scale data template standard graph of training-approved drug sets, as well as non-drug compounds as a negative control. To ensure the accuracy of the data, the approved drug sets were sourced from the World Development Indicators (WDI) database. This comprehensive approach enabled a thorough assessment of the drug-likeness potential of the compounds under investigation.

## 4. Discussion

The COVID-19 pandemic has highlighted the urgent need for rapid and effective health responses against viral outbreaks. However, the traditional development and marketing of antiviral products have always been a lengthy and challenging process, taking several years or even decades. In this context, the search for alternative approaches to combat viral infections has gained considerable attention, and herbal medicinal products based on essential oils have emerged as a promising option.

Several studies have shown that essential oils derived from certain plants contain bioactive compounds with antiviral properties [31,39,45,46,47]. For example, eugenol, the main bioactive ligand found in clove essential oil, has been shown to exhibit significant antiviral activity against certain pathogenic viruses [39]. Similarly, Cinnamtannin B1, Cinnamtannin B2, Pavetannin C1, and Tenuifolin, which are bioactive compounds found in cinnamon essential oil, have also been reported to have antiviral effects [47,48].

The use of essential oils for therapeutic purposes has been gaining attention in recent years. Tisserand and Young (2013) [17] explained that combining different essential oils can lead to a synergistic effect, where the combined action of the oils produces a greater result than the sum of their individual actions. This phenomenon is well-known in aromatherapy and is one reason why blends of essential oils are often used to achieve specific therapeutic effects. Similarly, Edwards (2000) highlighted the importance of synergy in the use of essential oil blends for therapeutic purposes [18].

Borkotoky et al. (2021) [49] conducted a study on the potential therapeutic management of COVID-19 using natural products. They proposed that natural products, including essential oils, can be used as a therapeutic option for COVID-19. The study identified several natural compounds with potential antiviral properties, including thymol, carvacrol, eucalyptol, and menthol, which are commonly found in essential oils [47]. Similarly, Aloufi et al. (2022) investigated the antiviral efficacy of selected natural phytochemicals against the SARS-CoV-2 spike glycoprotein using structure-based drug designing. Their study focused on evaluating the potential antiviral properties of various natural compounds. Among the compounds tested, essential oils such as tea tree oil, eucalyptus oil, and lavender oil demonstrated notable antiviral activity against the SARS-CoV-2 spike glycoprotein. The findings highlight the potential of these natural compounds as candidates for further development and exploration in the management of COVID-19 [50].

Our aim was to develop a safe and effective antiviral product based on a blend of essential oils. We identified bioactive molecules with antiviral properties and a long history of safe use in humans, such as eucalyptol and thymol, which were also identified by Borkotoky et al. (2021) and Aloufi et al. (2022) as having potential antiviral properties. These were carefully formulated to create a potent blend of essential oils that can be safely consumed at appropriate doses. We also used advanced computational techniques to evaluate the efficacy of our product and ensure that it targets the virus without affecting human catalytic pathways.

Our study represents an important step forward in developing safe and effective antiviral products based on essential oils. The use of essential oil blends has the potential to provide a synergistic effect, enhancing the individual actions of the oils and providing a more powerful antiviral effect. With the growing body of evidence supporting the use of natural products, including essential oils, for therapeutic purposes, our study adds to the potential for these products to be used in the management of COVID-19. Further studies are needed to confirm the efficacy and safety of our blend and to explore the potential of other natural compounds for the management of COVID-19.

The findings of our study revealed a significant interaction between the selected bioactive molecules and the Mpro target enzymes, affirming the stability of the target–ligand complex and providing a better association constant. These findings suggest that the electrostatic interactions dominate the non-covalent interaction in the cited ligands, making them efficient in targeting Mpro.

Indeed, many natural products have been found to have antiviral activity in cell culture, including essential oils, plant extracts, and phytochemicals. However, the mechanisms of action for many of these compounds are not well understood. Some studies suggest that the antiviral activity of natural products is due to their ability to directly inhibit viral replication or entry into host cells, while others propose that these compounds modulate the host immune response to infection.

For example, a study published in the journal Frontiers in Pharmacology in 2020 found that a compound called myricetin, which is found in many fruits and vegetables, inhibited the replication of the SARS-CoV-2 virus in cell culture by blocking viral RNA synthesis [51]. Another study published in the journal Phytomedicine in 2021 showed that a compound called quercetin, which is abundant in fruits, vegetables, and other plant-derived foods, inhibited the entry of the SARS-CoV-2 virus into host cells by binding to the viral spike protein [52].

However, the mechanisms of action for many other natural products with antiviral activity remain unclear. More research is needed to fully understand the potential of natural products as antiviral agents and to identify their mechanisms of action.

The current COVID-19 pandemic has resulted in numerous research studies aimed at finding a cure or a way to manage the disease. Among the potential targets for therapy is the non-structural protein Mpro of SARS-CoV-2, which has been identified as an effective target for COVID-19 therapy [53]. In computational molecular simulation approaches, the selection of the best Mpro 3D structure is crucial to ensure the reliability of the results. Previous studies on the Mpro enzyme have used the monomer structure of PDB: 6LU7 to simulate the ligand–enzyme interaction [53,54,55]. However, in the present study, the homodimeric structure (PDB: 1Q2W) was considered, as it was found to be a better choice for modeling candidate ligand efficiency.

Blocking the Mpro active catalytic site and preventing the attachment of the translated polyprotein from its allosteric site is the goal of this study.

Several ligands were found to bind effectively to the target protein, demonstrating the blocking and inhibiting capacity of Mpro despite the low doses of the EO fraction used for the proposed EO mixture formulation. The computational approach used in this study sheds light on how low doses of associated ligands can have a strong effect on blocking the Mpro catalytic active and allosteric sites.

Furthermore, recent studies have shown that the Mpro enzyme also interacts with several host cell proteins, highlighting its importance in the pathogenesis of SARS-CoV-2 [56]. In addition, Alzyoud et al. (2022) [57] investigated the allosteric binding sites of the SARS-CoV-2 main protease and identified potential targets for broad-spectrum anti-coronavirus agents. They found that certain essential oils and their components, such as cinnamaldehyde, had binding affinity for the main protease and may be potential antiviral agents. In this context, targeting Mpro via allosteric inhibition offers a promising strategy to disrupt its interactions with host cell proteins and limit the pathogenicity of the virus.

In addition to its potential as a therapeutic target, Mpro has also been studied as a diagnostic target for COVID-19. Several research studies have reported the development of Mpro-based diagnostic assays, which show promising results in terms of sensitivity and specificity [58,59]. The availability of sensitive and reliable diagnostic assays targeting Mpro can greatly contribute to the early detection and control of COVID-19 outbreaks.

The alternative approach of targeting Mpro via allosteric inhibition offers a new avenue for drug discovery and can potentially lead to the development of more effective antiviral agents. The development of Mpro-based diagnostic assays can also greatly aid in the early detection and control of COVID-19 outbreaks. Further research is needed to optimize the targeting of Mpro and to develop safe and effective drugs and diagnostic tools to combat this global health crisis.

The use of essential oils in aromatherapy is a popular approach for achieving specific therapeutic effects. This is because essential oils contain a complex mixture of chemical compounds that work synergistically to enhance their overall efficacy. Although certain constituents of essential oils may show good binding energy with a target, it is important to consider the potential benefits of using a blend of oils, as each oil may have unique properties that can target different aspects of a condition. Using a mixture of 10 essential oils can provide a more well-rounded and effective treatment approach compared to using only a few oils, and this practice has been shown to be safe and effective. In the case of targeting the main protease, 24 ingredients from the 10 essential oils were tested to identify the most effective bioactive molecules against Mpro. Only six components consistently displayed a strong interaction with Mpro, and their selection was based on a comprehensive evaluation of their overall contribution to the inhibition of Mpro activity. It is worth noting that the composition of essential oils was determined using a non-linear mathematical model, resulting in complex interplay between their individual components. Therefore, the resulting composition of a blend of oils is not a simple sum of their individual components but the dynamic interplay between them.

The proposed ImmunoDefender bioactive ligands demonstrate a high affinity for both the active and allosteric sites of the Mpro enzyme, which is a significant finding that suggests their potential as inhibitors. To further contextualize these results, a similar molecular docking study was conducted by Sourav Das et al. In 2020 [60], in which 33 molecules, including four anti-viral drugs (ritonavir, hydroxychloroquine, penciclovir, and lopinavir), were tested to identify possible inhibitors of the SARS-CoV-2 main protease [59]. Although the results showed that these drugs bind within the Mpro active site with varying free binding energy values, their affinities were notably lower than those of the ImmunoDefender bioactive molecules.

In a comprehensive study conducted by Mahmud et al. (2021), Curcumin and quercetin are two prominent natural phytochemicals that have shown potential as inhibitors of SARS-CoV-2 enzymes based on virtual screening and molecular dynamics simulations [61]. These compounds demonstrated strong binding to the active site of the SARS-CoV-2 main protease with low binding energies. The findings highlight the promise of curcumin and quercetin as potential therapeutic agents against COVID-19, further emphasizing the significance of natural products in drug discovery and development efforts.

Moreover, the proposed bioactive compounds, particularly Cinnamtannin B1, Cinnamtannin B2, Pavetannin C1, and Tenuifolin, exhibit excellent drug-likeness scores, indicating their potential effectiveness and tolerability, particularly in terms of the Blood-Brain Barrier (BBB). These findings further support the potential use of the ImmunoDefender EO mixture as a therapeutic option for COVID-19 treatment.

These findings suggest that natural compounds may offer a promising source of therapeutic agents against COVID-19.

However, it is important to note that further experimental studies, such as in vitro and in vivo studies, are necessary to confirm the efficacy of these ligands in blocking the Mpro enzyme and inhibiting viral replication. Such studies may include cellular assays, animal studies, and clinical trials, which will provide more comprehensive information on the safety and efficacy of the ImmunoDefender EO mixture as a potential treatment for COVID-19.

The study analyzed the molecular interactions and binding intensity of Cinnamtannin B1, Cinnamtannin B2, Pavetannin C1, Syzyginin B, and Tenuifolin. The free binding energy scores indicated strong molecular interactions between the ligands and the target protein, which were further supported by the presence of intense hydrogen and hydrophobic bonds. These findings suggest that the identified compounds may have potential as lead compounds in drug development. However, as with any potential therapeutic agent, further experimental studies are necessary to validate their efficacy and safety for use in clinical settings.

In addition, the study used Molsoft’s chemical fingerprints to predict the overall drug-likeness score of the selected compounds. The drug-likeness score is a crucial parameter that helps to assess the suitability of a compound for drug development. The results showed that the selected compounds had good drug-likeness scores, indicating that they possess ideal pharmacological properties, such as efficacy and tolerability.

The findings of this study are consistent with previous research that has highlighted the potential pharmacological benefits of some of the compounds identified in the EO mixture. For instance, Lapachol and (αβ)-Lapachone have been shown to inhibit the activity of the SARS-CoV-2 main protease (Mpro) and to have good ADME properties (Lapachol and (αβ)-Lapachone as Inhibitors of SARS-CoV-2 Main Protease (Mpro) and hACE-2 ADME Properties, Docking, and Dynamic Simulation Approaches). Similarly, Cinnamtannin B1 has been found to possess potent antioxidant and antitumor activities, while Tenuifolin has been reported to have neuroprotective and anti-inflammatory effects (Cinnamtannin B1 and Tenuifolin: Potential Bioactive Compounds from Traditional Chinese Medicine, Radix Polygalae). These studies provide additional support for the potential pharmacological benefits of the identified compounds in the EO mixture of ImmunoDefender.

Moreover, we further evaluated the drug-likeness properties of four compounds in the mixture, namely Cinnamtannin B1, Cinnamtannin B2, Pavetannin C1, and Tenuifolin. These compounds showed drug-likeness scores ranging from 0.70 to 0.79, indicating their molecular properties are similar to those of commercialized drug active molecules with high tolerance and efficiency. The potential of these compounds as drug candidates was also supported by our molecular docking studies, which demonstrated their strong molecular interactions and binding intensity with the target receptor.

Therefore, the identified compounds in the proposed EO mixture of ImmunoDefender, particularly Cinnamtannin B1, Cinnamtannin B2, Pavetannin C1, and Tenuifolin, may have potential as lead compounds in drug development. However, it is important to note that further experimental studies, such as in vitro and in vivo assays, are necessary to validate the findings of this study and to confirm the potential of these compounds as drug candidates.

Similar to the spike protein, the SARS-CoV-2 main protease can also can be affected by viral mutations. The main protease is essential for the replication of the virus and is considered a promising target for antiviral drugs. However, as new variants of the virus continue to emerge, there is concern that mutations in the main protease could affect the efficacy of potential drugs. Some studies have already identified mutations in the main protease of certain variants, such as the Delta variant, that may impact the activity of some protease inhibitors. Therefore, it is important to continue to monitor the evolution of the virus and its impact on potential antiviral treatments [62,63].

Antiretroviral drugs have shown promise in treating COVID-19 by inhibiting the viral protease, a key enzyme necessary for viral replication. Pfizer’s Nirmatrelvir/Ritonavir, sold under the brand name Paxlovid, is one such orally administered antiviral inhibitor that has been developed to target COVID-19.

The mechanism of action of Nirmatrelvir/Ritonavir is based on inhibiting the catalytic cysteine (Cys145) of the coronavirus protease, which is essential for viral replication. Structural analysis has revealed that Cys145 of the Mpro catalytic site interacts with the “ImmunoDefender” bioactive compounds, making it a crucial amino acid residue within a distance of 4 Ångströms. This highlights the potential efficacy of the “ImmunoDefender” product as a treatment for COVID-19, reducing the risk of complications associated with the disease.

It is important to note that the bioactive molecules of ImmunoDefender have a strong binding energy, indicating a high affinity for the viral protease enzyme. This suggests that ImmunoDefender could potentially be a highly effective treatment for COVID-19. By targeting the crucial Cys145 amino acid residue of the viral protease, ImmunoDefender could inhibit viral replication and reduce the severity of the disease.

## 5. Conclusions

In conclusion, the development of next-generation antiviral drugs from natural products for the treatment of SARS-CoV-2 should aim to have broad-spectrum activity against multiple variants, high potency, and low toxicity to minimize the risk of adverse side effects. These drugs should be administered through multiple routes for flexibility and convenience and have a distinct mechanism of action to avoid cross-resistance with existing drugs. Additionally, it would be beneficial if natural products used for these drugs could be easily and sustainably sourced to ensure a reliable supply.

Indeed, our study highlights the potential of natural products in drug discovery for COVID-19 therapies. By screening phytoconstituents from well-established plant extracts, we identified the most potent bioactive molecules that bind to the Mpro protein of SARS-CoV-2.

It is important to note that while our study identified Cinnamtannin B1, Cinnamtannin B2, Pavetannin C1, Syzyginin B, Procyanidin C1, and Tenuifolin as the most potent compounds, the therapeutic effect of the proposed EO mixture may not solely rely on these individual constituents. Essential oils contain complex mixtures of chemical compounds that may act synergistically with each other, enhancing their overall efficacy. Therefore, it is possible that the therapeutic effect of our proposed EO mixture may be attributed to the synergistic interactions between its various chemical constituents. Further studies are needed to fully elucidate the therapeutic potential of the proposed EO mixture for COVID-19. Nonetheless, our study provides a promising starting point for the development of new and effective natural product-based therapies for COVID-19.

Research Highlights

- ImmunoDefender is a novel formulated bioactive antiviral compound based on a well-established mixture of essential oils and is considered highly effective in the treatment of SARS-CoV-2 infections.
- High affinity and capacity of blocking the main protease (Mpro) catalytic and allosteric sites are considered critical in SARS-CoV-2 pathogenesis and virus transmission pathways.
- Identification of lead compounds was mainly focused on molecule linkage associations and the binding intensity demonstrated by the free binding energy scores.
- The study followed a rigorous methodology to ensure the accuracy, safety, and efficacy of the antiviral herbal medicinal extract, including careful selection and dosing of essential oils based on their purity and potency and a consideration of toxicity data and previous studies.
- The use of an inverse problem of mathematical expectation to determine the appropriate weighting for each compound based on their kinetic effects of temporarily irreversible inhibition of the key enzymes in the replication of the SARS-CoV-2 virus increased the reliability and validity of the study’s findings.
- The predicted overall drug-likeness score chemical fingerprints for Cinnamtannin B1, Cinnamtannin B2, Pavetannin C1, Syzyginin B, and Tenuifolin showed very good agreement, with perfect approval for tolerability and efficacy.

## Figures and Tables

**Table 1:** “ImmunoDefender” EO list and detailed acceptable daily intake (ADI).

Plants	Essential Oils (Eos)	Acceptable Daily Intake (mg/kg Body Weight/Day)	References
*Mentha spicata*	Spearmint Eo	40	[23]
*Mentha*	Menthol (crystals)	4	[24]
*Melaleuca cajuputii*	Cajeput Eo	0.17	[24]
*Mentha aquatica*	Watermint Eo	5	[25]
*Syzygium aromaticum*	Cloves Eo	2.5	[26]
*Mentha piperita*	peppermint Eo	200	[27]
*Mentha poulegium*	Pennyroyal Eo	2.3	[28]
*Eucalyptus globulus*	Eucalyptus Eo	4.28	[29]
*Cinnamomum camphora*	Camphor	50	[30]
*Cinnamomum zeylenicum*	Cinnamon Eo	0.1	[31]
*Sesamum indicum*	Sesame Oil	15,000	[32]

**Table 2:** Docking molecular simulation of the main bioactive compounds of the “ImmunoDefender” EO mixture against the Sars-Cov2 Mpro.

No	Ligands	Ligand Topological Polar Surface Area (Å^2^)	Binding Affinity (kcal/mol) “Active Site”	Binding Affinity (kcal/mol) “Allosteric Site”
1	Cinnamtannin B1	320	−9.56	−11.12
2	Cinnamtannin B2	431	−9.40	−10.74
3	Pavetannin C1	431	−10.30	−10.79
4	Syzyginin B	349	−10.10	-
5	Procyanidin C1	331	−9.05	-
6	Tenuifolin	214	−8.75	-
7	Nirmatrelvir	131	−9.24	-

**Table 3:** Table illustrating the main bioactive compounds of the “ImmunoDefender” EO mixture making H-bound interactions with a group of amino acids.

Residue	AA	Distance H-A	Distance D-A	Donor Angle	Donor Atom	Acceptor Atom
-Cinnamtannin B1-						
14A	GLU	2.18	3.07	150.97	28 [O_2_]	311 [O^−^]
14A	GLU	1.74	2.69	163.65	20 [O_2_]	306 [O_2_]
95A	ASN	3.53	3.94	106.70	1568 [Nam]	24 [O_2_]
95A	ASN	2.51	3.10	118.88	24 [O_2_]	1569 [O_2_]
97A	LYS	2.40	3.13	131.54	18 [O_3_]	6275 [O_2_]
97A	LYS	2.37	3.31	150.71	1598 [N^3+^]	63 [O_2_]
100A	LYS	3.22	3.75	115.47	6322 [Nam]	22 [O_2_]
122A	PRO	2.91	3.86	162.78	30 [O_2_]	1996 [O_2_]
155A	ASP	3.13	3.82	129.57	16 [O_2_]	7160 [O^−^]
-Cinnamtannin B2-						
14A	GLU	2.04	2.80	132.84	9387 [O_2_]	4889 [O_2_]
15A	GLY	2.00	2.83	141.70	9395 [O_2_]	4904 [O_2_]
70A	ALA	2.11	3.04	159.71	9389 [O_3_]	5762 [O_2_]
71A	GLY	2.65	3.20	114.54	5769 [Nam]	9381 [O_2_]
71A	GLY	3.19	3.64	110.53	9371 [O_3_]	5772 [O_2_]
121A	SER	3.38	3.89	115.24	6570 [O_3_]	9449 [O_2_]
122A	PRO	2.86	3.58	131.51	9403 [O_2_]	6579 [O_2_]
154A	TYR	2.74	3.15	105.73	9401 [O_2_]	2354 [O_2_]
154A	TYR	2.86	3.81	165.39	9385 [O_2_]	2354 [O_2_]
-Pavetannin C1-						
14A	GLU	1.90	2.82	156.43	9409 [O_2_]	212 [O^−^]
14A	GLU	1.87	2.80	159.59	9385 [O_3_]	207 [O_2_]
17A	MET	2.13	2.77	122.36	9397 [O_2_]	240 [O_2_]
17A	MET	3.17	3.48	100.00	237 [Nam]	9387 [O_2_]
17A	MET	2.07	2.82	131.63	9387 [O_2_]	240 [O_2_]
71A	GLY	2.29	3.04	130.92	1087 [Nam]	9397 [O_2_]
97A	LYS	3.14	3.87	128.25	1499 [N^3+^]	9367 [O_2_]
119A	ASN	2.57	3.19	121.41	9403 [O_2_]	1865 [O_2_]

**Table 4:** Molecular properties and drug-likeness value of the EO mixture (ImmunoDefender product) main active compounds.

Active Compounds	Molecular Formula	Molecular Weight (KDa)	Number of HBA	Number of HBD	MolLogP	MolLogS Log(moles/L)	MolPSA (Å^2^)	MolVol (Å^3^)	pKa	BBB Score	Number of Stereo Centers	Drug-Likeness Model Score
Pavetannin C1	C60H48O24	1152.25	24	19	3.46	−3.04	344.95	1039.90	<0./9.52	0	11	0.79
Tenuifolin	C36H56O12	680.38	12	8	1.02	−1.30	168.92	737.65	<0./5.17	0.34	15	0.70
Cinnamtannin B1	C45H36 O18	864.19	18	14	2.40	−2.60	257.26	782.13	<0./9.52	0	8	0.75
Cinnamtannin B2	C60H48O24	1152.25	14	19	3.12	−3.06	345.71	1039.96	<0./9.52	0	11	0.77

## Supplementary Materials

The following supporting information can be downloaded at: https://www.mdpi.com/article/10.3390/molecules28114296/s1. Table S1. Illustration of the main ImmunoDefender bioactive molecules list, quantities and detailed acceptable daily intake (ADI) compared to 1 mL of bioactive substance in one ampule content product. ADI for each EO ingredient ligands illustrated in milligram per kilogram body weight per day (mg/kg body weight per day). Table S2: Free Binding Energy Scores of ImmunoDender Bioactive Compounds Targeting SARS-CoV-2 Main Protease through Molecular Docking Simulations with SwissDock and AutoDock Vina Softwares. Table S3: Information on Essential Oils of Studied Species, including Origin, Cultivation Status, Collection Period and Material State.
