# Supplementary material for: Docking-Based Evidence for the Potential of ImmunoDefender: A Novel Formulated Essential Oil Blend Incorporating Synergistic Antiviral Bioactive Compounds as Promising Mpro Inhibitors against SARS-CoV-2
Source: Molecules. 2023 May 24;28(11):4296. doi: 10.3390/molecules28114296 (PMC10254185; doi:10.3390/molecules28114296)
Supplement: Supplementary file 1 [file molecules-28-04296-s001.zip › molecules-2364543-supplementary.pdf]

Supplementary data

Table S1. Illustration of the main ImmunoDefender bioactive molecules list, quantities and detailed acceptable daily intake (ADI) compared to 1 ml of bioactive substance in one ampule content product. ADI for each EO ingredient ligands illustrated in milligram per kilogram body weight per day (mg/kg body weight per day).

Ingredient	Total substance ingredients (mg)	Acceptable daily intake (ADI) mg/kg body weight per day	References
Carvone	33	1.25	[38]
Limonene	25.08	1.5	[39]
Menthol	139.41	4	[40]
Pipéritone	64	No safety concern	[28]
α-Phellandrène	32	No safety concern	[28]
Ocimène	5.67	4	[42]
Pulegone	13.92	0.1	[43]
Menthone	14	No safety concern	[28]
1,8 cineole	102.16	No safety concern	[28]
Eugenol	49.56	1	[44]
Beta caryophyllène	12.48	No safety concern	[39]
α-humulene	1	0.11	[45]
Eugenyl acetate	0.72	No safety concern	[28]
Neo-menthol	8.2	No safety concern	[28]
Menthylacetate	6.05	No safety concern	[28]
α-terpineol	27.85	No safety concern	[28]
A-terpinyl acetate	9.71	No safety concern	[28]
α-pinene	1.56	No safety concern	[28]
Cinnamaldéhyde	46.08	1.25	[35]
Cinnamyl acetate	1.86	No safety concern	[28]
Cinnamtannin B1	not specified	not specified	
Cinnamtannin B2	not specified	not specified	
Pavetanine C1	not specified	not specified	
Tenuifoline	not specified	not specified	

Table S2 : Free Binding Energy Scores of ImmunoDender Bioactive Compounds Targeting SARS-CoV-2 Main Protease through Molecular Docking Simulations with SwissDock and AutoDock Vina Softwares.

Ingredient	Free binding energy score of docking simulated with Mpro via SwissDock (kcal/mol)	Free binding energy score of docking simulated with Mpro via AutoDock (kcal/mol)
Carvone	- 4.8	- 4.5
Limonene	- 4.7	- 4.9
Pipéritone	-5	-4.7
α-Phellandrène	-4.6	-4.5
Ocimène	- 4.9	- 4.8
Pulegone	-4.7	-4.2
Menthone	-5.2	-4.8
1,8 cineole	- 4.8	- 5.1
Eugenol	- 6.8	- 6.5
Cinnamtannin B1	-9.5	- 8.6
Cinnamtannin B2	-9.4	- 8.4
Beta caryophyllene	-7	-6.7
α-humulene	-4.3	-4.7
Syzyginin B	-10.1	-9.7
Eugenyl acetate	-6.7	-6.1
Pavetanine C1	-10.3	-9.1
menthol	-6.1	-5.7
Procyanidin C1	-9.05	-8.1
Menthylacetate	- 4.7	- 4.9
α-terpineol	-5	-4.6
Comphor	-6.8	-6.1
α-pinene	-4.7	-4.9
Cinnamaldéhyde	-7.4	-6.9
Tenuifoline	-8.7	-8.1

Table S3 : Information on Essential Oils of Studied Species, including Origin, Cultivation Status, Collection Period and Material State:

Essential Oils of Studied Species	information about the studied species essential oils
Mentha spicata	The essential oil of Mentha spicata (Spearmint) is obtained by steam distillation of the leaves and flowering tops of the plant. The plant is often cultivated for its essential oil and is widely distributed in many countries. The period and year of collection, as well as whether the material used was fresh or dried, would depend on the specific source of the essential oil.
Mentha aquatica	The essential oil of Mentha aquatica (Water mint) is obtained by steam distillation of the leaves and flowering tops of the plant. The plant is often found growing wild in damp areas such as marshes and along stream banks. The period and year of collection, as well as whether the material used was fresh or dried, would depend on the specific source of the essential oil.
Syzygium aromaticum	The essential oil of Syzygium aromaticum (Clove) is obtained by steam distillation of the dried flower buds of the plant. The plant is often cultivated in tropical countries and the essential oil is widely used in the perfume and flavor industries. The period and year of collection, as well as the state of the material used, would depend on the specific source of the essential oil.
Mentha piperita	The essential oil of Mentha piperita (Peppermint) is obtained by steam distillation of the leaves and flowering tops of the plant. The plant is often cultivated for its essential oil and is widely distributed in many countries. The period and year of collection, as well as whether the material used was fresh or dried, would depend on the specific source of the essential oil.
Mentha pulegium	The essential oil of Mentha pulegium (Pennyroyal) is obtained by steam distillation of the aerial parts of the plant. The plant is often found growing wild in damp areas such as marshes and along stream banks. The period and year of collection, as well as

	whether the material used was fresh or dried, would depend on the specific source of the essential oil.
Eucalyptus	The essential oil of Eucalyptus is obtained by steam distillation of the leaves and twigs of various species of the Eucalyptus tree. The trees are often cultivated in subtropical and tropical regions and are also found growing wild. The period and year of collection, as well as whether the material used was fresh or dried, would depend on the specific source of the essential oil.
Cinnamomum zeylanicum	The essential oil of Cinnamomum zeylanicum (Cinnamon) is obtained by steam distillation of the bark of the tree. The tree is often cultivated in tropical regions and is also found growing wild. The period and year of collection, as well as the state of the material used, would depend on the specific source of the essential oil.
Melaleuca cajuputi	The essential oil of Melaleuca cajuputi (Cajeput) is obtained by steam distillation of the leaves and twigs of the tree. The tree is often found growing wild in subtropical and tropical regions. The period and year of collection, as well as whether the material used was fresh or dried, would depend on the specific source of the essential oil.
Cinnamomum camphora	is obtained by steam distillation of the wood of the tree. The tree is often cultivated in subtropical and tropical regions and is also found growing wild. The period and year of collection, as well as the state of the material used, would depend on the specific source of the essential oil. A voucher specimen may be used to verify the identity of the plant material used to obtain the essential oil.
